# The influence of spontaneous and visual activity on the development of direction selectivity maps in mouse retina

**DOI:** 10.1016/j.celrep.2021.110225

**Published:** 2022-01-11

**Authors:** Alexandre Tiriac, Karina Bistrong, Miah N. Pitcher, Joshua M. Tworig, Marla B. Feller

**Affiliations:** 1 Department of Molecular and Cell Biology, University of California, Berkeley, Berkeley, CA 94720, USA; 2 Helen Wills Neuroscience Institute, University of California, Berkeley, Berkeley, CA 94720, USA; 3 Lead contact

## Abstract

In mice, retinal direction selectivity is organized in a map that aligns to the body and gravitational axes of optic flow, and little is known about how this map develops. We find direction selectivity maps are largely present at eye opening and develop normally in the absence of visual experience. Remarkably, in mice lacking the beta2 subunit of neuronal nicotinic acetylcholine receptors (β2-nAChR-KO), which exhibit drastically reduced cholinergic retinal waves in the first postnatal week, selectivity to horizontal motion is absent while selectivity to vertical motion remains. We tested several possible mechanisms that could explain the loss of horizontal direction selectivity in β2-nAChR-KO mice (wave propagation bias, FRMD7 expression, starburst amacrine cell morphology), but all were found to be intact when compared with WT mice. This work establishes a role for retinal waves in the development of asymmetric circuitry that mediates retinal direction selectivity via an unknown mechanism.

## In brief

The direction selectivity of motion is first computed in the retina. In adults, four directions are preferentially represented and align to the axes of optic flow. Tiriac et al. show that this organization develops normally in dark-reared mice but not in mice that exhibit perturbed spontaneous activity during development.

## Graphical Abstract

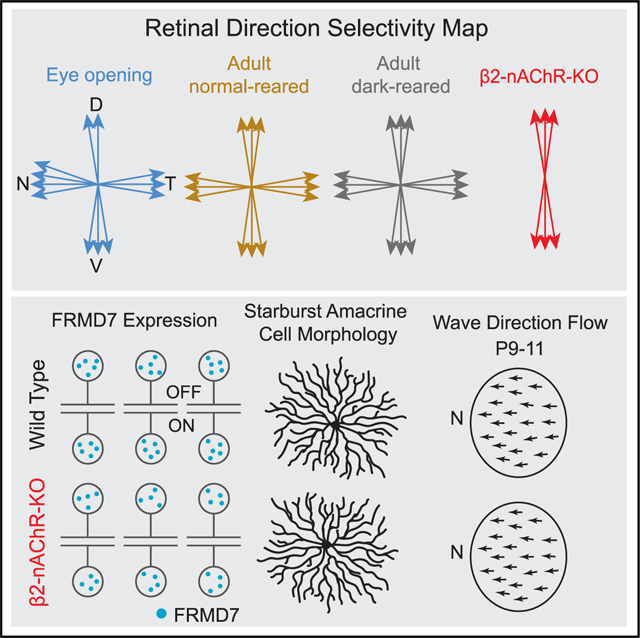

## INTRODUCTION

Detecting the direction of visual motion, whether generated by self-motion or by objects moving within the visual field, is critical for everyday behavior. However, not all directions of motion are equally represented in the visual system. In many species, direction selectivity is first manifested in the retina (Baden et al., 2020). Direction-selective retinal ganglion cells (DSGCs) fire more action potentials in response to visual stimuli moving in one direction, called the preferred direction, than visual stimuli moving in the opposite direction, called the null direction (Barlow and Levick, 1965). In many mammals, including mice, the preferred directions of DSGCs cluster in four groups along two axes. The relative orientations of these axes vary with retinal location—following the axes of optic flow and converging onto points called singularities. Whereas the preferred directions of nasal- and temporal-preferring DSGCs follow the body axis, the preferred directions of dorsal- and ventral-preferring DSGCs follow the gravitational axis (Sabbah et al., 2017).

Several studies support the idea that the presence of directional visual stimulation instructs development of direction selectivity in the visual cortex: horizontal motion detection develops after eye opening when animals become mobile (Jenks and Shepherd, 2020), visual deprivation leads to fewer direction-selective cells (Li et al., 2006), and exposure to directional motion at particular ages increases the proportion of direction-selective cells (Li et al., 2008; Roy et al., 2020). In the retina, the very fact that direction selectivity follows the axes of optic flow, which animals experience as soon as they can explore their environment, suggests that visual experience could play a role in setting up this map. Alternatively, retinal waves, which spontaneously occur during development, exhibit a propagation bias that has been implicated in setting up direction selectivity in the superior colliculus (Ge et al., 2021). In this study, we assess the relative roles of visual experience and retinal waves in the development of the direction selectivity map.

## RESULTS

**Figure 1.**
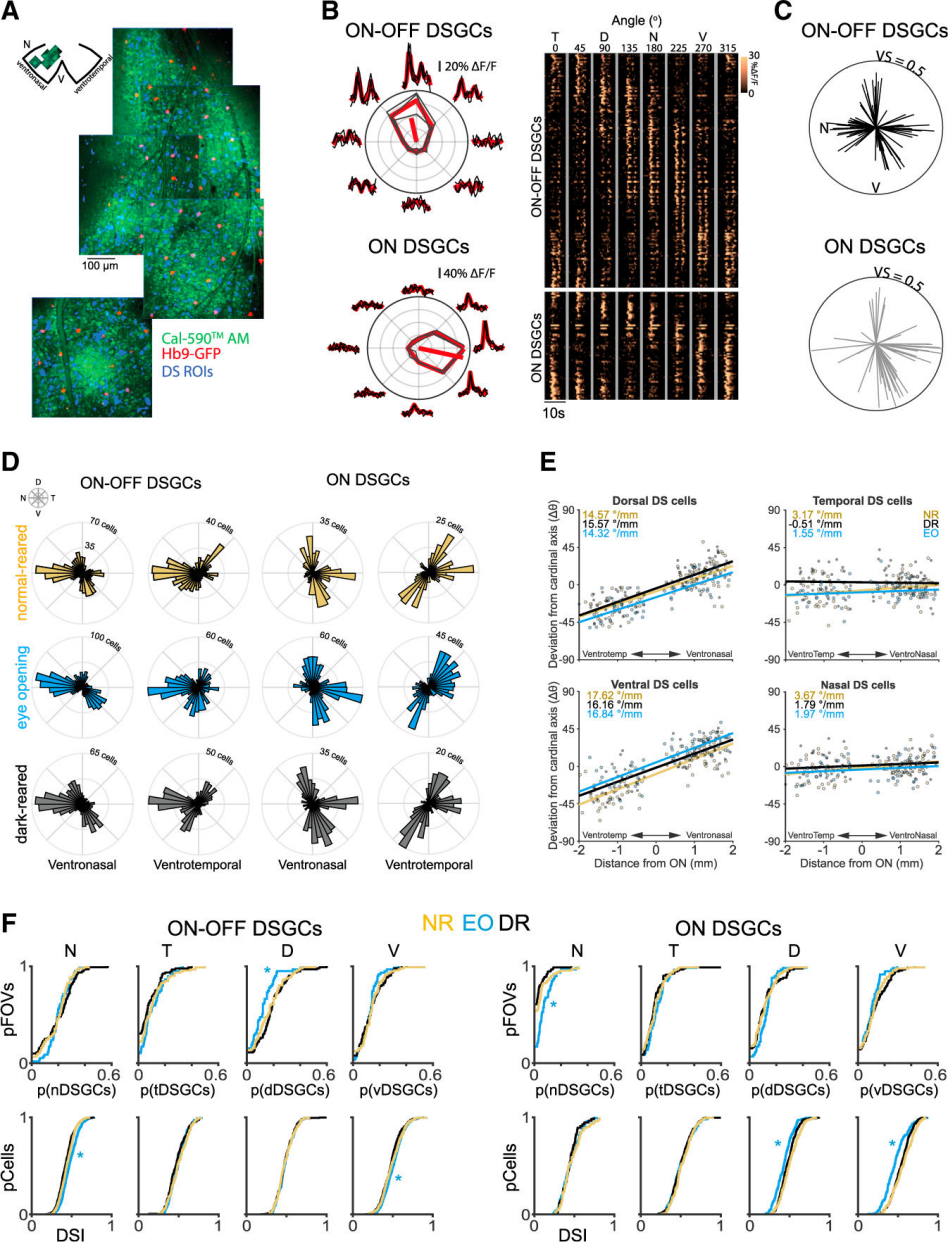
The direction selectivity map develops independent of visual experience (A) Schematic showing mapping experiment of direction selectivity (DS) across a quadrant of the retina. (B) Left: tuning curve of an example ON-OFF and ON DSGC. Right: heatmap of average responses to moving bars of ON-OFF and ON DSGCs identified from all fields of view (FOVs) in (A). Each row is a different cell and is sorted based on preferred direction. (C) Local DS map of the analyzed FOV shown in (A) for ON-OFF DSGCs (black) and ON DSGCs (gray). For each DS map, each vector is a single DSGC, with its direction representing the cell’s preferred direction and the length representing the strength of tuning. VS, vector sum. (D) ON-OFF and ON DS maps of normal-reared (NR) adults (yellow; n = 2,041 DSGCs across 10 mice), eye opening (EO) (blue; n = 2472 DSGCs across 9 mice), and dark-reared (DR) adults (black; n = 2261 DSGCs across 8 mice) in ventronasal and ventrotemporal retina. (E) Rate of change of a functional group’s preferred direction (dorsal, ventral, temporal, nasal) as a function of distance from the optic nerve (ON) in the ventronasal and ventrotemporal axis. Each data point is a FOV. (F) Top: cumulative proportions of DSGCs within different functional subgroups (N, nasal; T, temporal; D, dorsal; V, ventral) across FOVs for ON-OFF (left) and ON DSGCs (right) and across different experimental groups. Bottom: proportions of cells that exhibit different various DSI. *Different from NR adult, p < 0.01 (ANOVA followed by Tukey-Kramer post hoc test).

To assess whether visual experience influences the establishment of this map, we performed two-photon calcium imaging over large areas of ventronasal and ventrotemporal retinas (Figure 1A). We functionally classified DSGCs depending on whether they responded to both the onset and offset of the light (ON-OFF) or just to the onset of the light (ON) (Figure 1B). In a subset of experiments, we used transgenic mice where ventral-preferring (Hb9-GFP) or nasal-preferring (Drd4-GFP) DSGCs were labeled with GFP. ON-OFF and ON direction selectivity maps looked very similar, with the exception that ON maps had negligible numbers of nasal-preferring DSGCs (Figure 1C).

In contrast to previous studies (Bos et al., 2016; Chan and Chiao, 2013), we found that direction selectivity maps were well established by eye opening (Figure 1D). In addition, these maps were indistinguishable between normal-reared and dark-reared adult mice. To compare direction selectivity maps across these three conditions, we functionally clustered ON-OFF DSGCs and ON DSGCs into four groups according to their preferred direction (see STAR Methods; Figure S1) (Sabbah et al., 2017). In all three experimental groups, the preferred directions of vertical-preferring (dorsal and ventral) DSGCs changed as a function of retinal location (Figure 1E), converging toward the ventral pole (Figure S2), as described previously (Sabbah et al., 2017). At eye opening, there were fewer dorsal-preferring ON-OFF DSGCs and more nasal-preferring ON DSGCs compared with the adult, with this latter difference potentially due to an immature OFF response of ON-OFF DSGCs at eye opening (Hilgen et al., 2017; Rosa et al., 2016). Vertical-preferring ON DSGCs exhibited a small but significantly lower direction selectivity index (DSI) at eye opening (DSI = 0.44 ± 0.01; mean ±95% CI) than at adulthood (DSI = 0.53 ± 0.01; mean ±95% CI). Moreover, there were no significant differences in the proportion of DSGCs that fall within each functional group or in tuning strength between adult normal- and dark-reared mice (Figures 1F and S3). These results were similar for genetically identified subsets of ON-OFF DSGCs (Figures S2 and S4 Hb9 and Drd4 data). These results therefore indicate that vision plays a minimal role in the establishment or maintenance of retinal direction selectivity maps. We hypothesize that our previous results indicating reduced clustering of preferred directions at eye opening and after dark rearing (Bos et al., 2016) were due to under-sampling and pooling of data across the entire retina, therefore not accounting for local differences in the relative orientation of preferred directions (Figure S5).

We next explored whether spontaneous activity plays a role in the development of direction selectivity maps. Before eye opening, the primary driver of retinal activity stems from retinal waves, which are spontaneously produced bursts of activity that propagate across the retinal ganglion cell layer (Maccione et al., 2014; Meister et al., 1991). During the first postnatal week, retinal waves are mediated by cholinergic circuits and exhibit a propagation bias in the direction of forward optic flow (Ackman et al., 2012; Elstrott and Feller, 2010; Stafford et al., 2009). In mice where the β2 subunit of the nicotinic acetylcholine receptor is genetically ablated (β2-nAChR-KO), retinal waves are severely disrupted (Burbridge et al., 2014; Stafford et al., 2009). Moreover, β2-nAChR-KO mice lack a horizontal optokinetic reflex (Wang et al., 2009), which is dependent on retinal direction selectivity (Shi et al., 2017; Yoshida et al., 2001). We therefore assessed direction selectivity maps in the retinas of β2-nAChR-KO mice.

**Figure 2.**
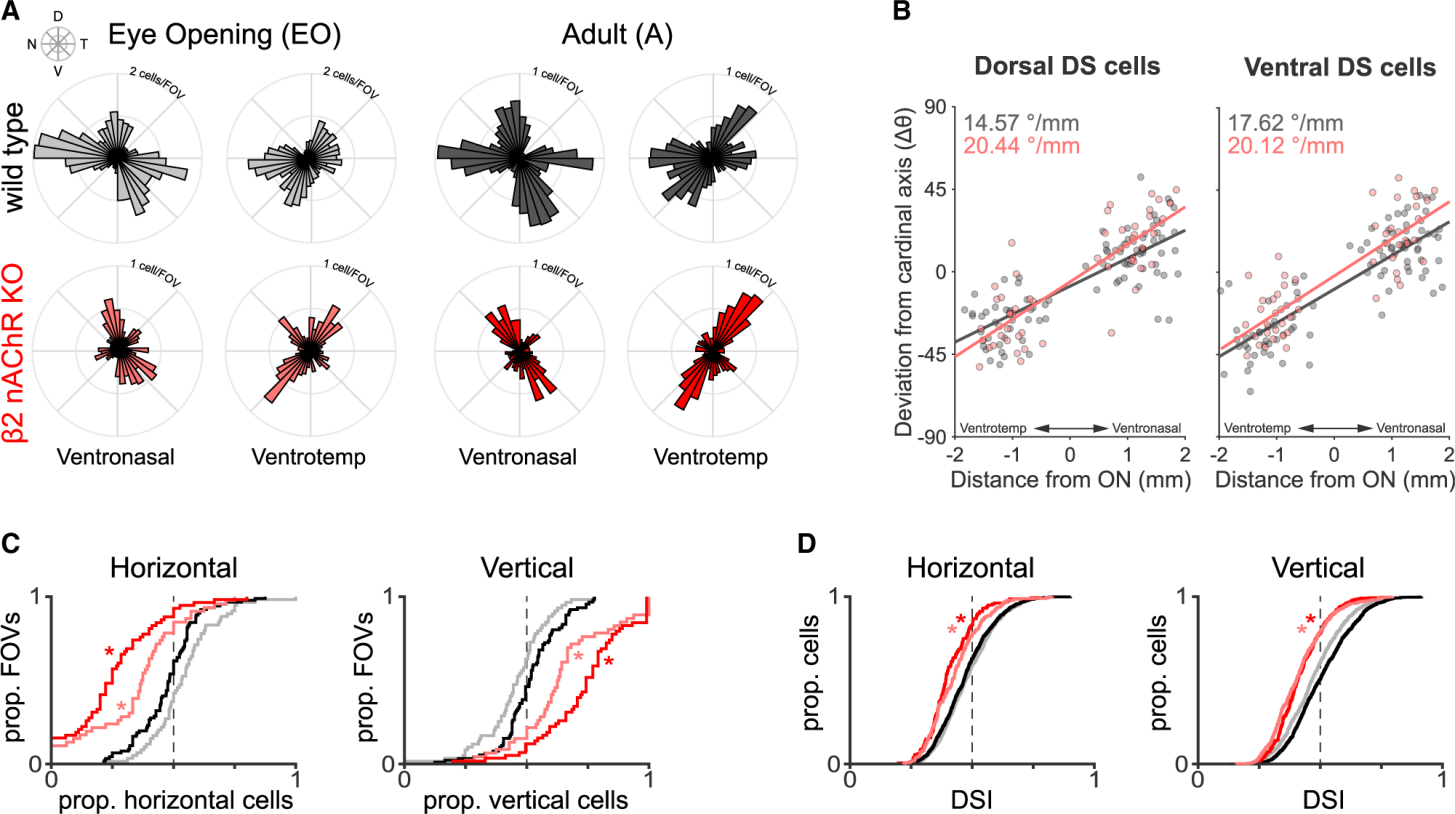
Retinal waves set up the horizontal component of the direction selectivity map (A) Direction selectivity (DS) maps of WT (gray/black) and β2-nAChR-KO mice (pink/red) at eye opening (gray/pink) and adulthood (black/red) in ventronasal and ventrotemporal retinas. Here, ON-OFF and ON DSGCs are combined into one plot because the effect occurs in both subtypes (see Figure S6). N = 741 for EO β2-nAChR-KO mice DSGCs across 5 mice; N = 781 for adult β2-nAChR-KO mice DSGCs across 8 mice. (B) Rate of change of a functional group’s preferred direction as a function of distance from the optic nerve (ON) for adult WT (black) and β2-nAChR-KO (red) mice. (C) Cumulative proportions of horizontal- or vertical-preferring ON-OFF and ON DSGCs across fields of view. *Denotes significant difference in proportion in β2-nAChR-KO from both EO WTs and adult WTs, p < 0.01 (ANOVA followed by Tukey-Kramer post hoc test). Note: horizontal and vertical proportion data sum to 1 and are both shown for clarity. (D) Direction selectivity index (DSI) values of horizontal- or vertical-preferring ON-OFF and ON DSGCs. *Different from both EO WTs and adult WTs, p < 0.01 (ANOVA followed by Tukey-Kramer ost hoc test).

At eye opening, β2-nAChR-KO mice exhibited a dramatic reduction in selectivity to horizontal motion (nasal and temporal) (Figures 2A and 2C) and a slight reduction in the tuning strength of both horizontal- and vertical-preferring DSGCs (Figure 2D). This absence of horizontal direction selectivity persisted into adulthood (Figure 2A). These results were similar when we analyzed individual functional subtypes of ON-OFF and ON DSGCs (Figure S6). In addition, we observed a reduction in the number of vertical-preferring DSGCs, but those that remained still mapped along the axes of optic flow. Moreover, there was a small but significant increase in the magnitude of variation along the vertical axes compared with vertical-preferring DSGCs in wild-type (WT) mice (Δ ~ 3–5°/mm; Figure 2B).

**Figure 3.**
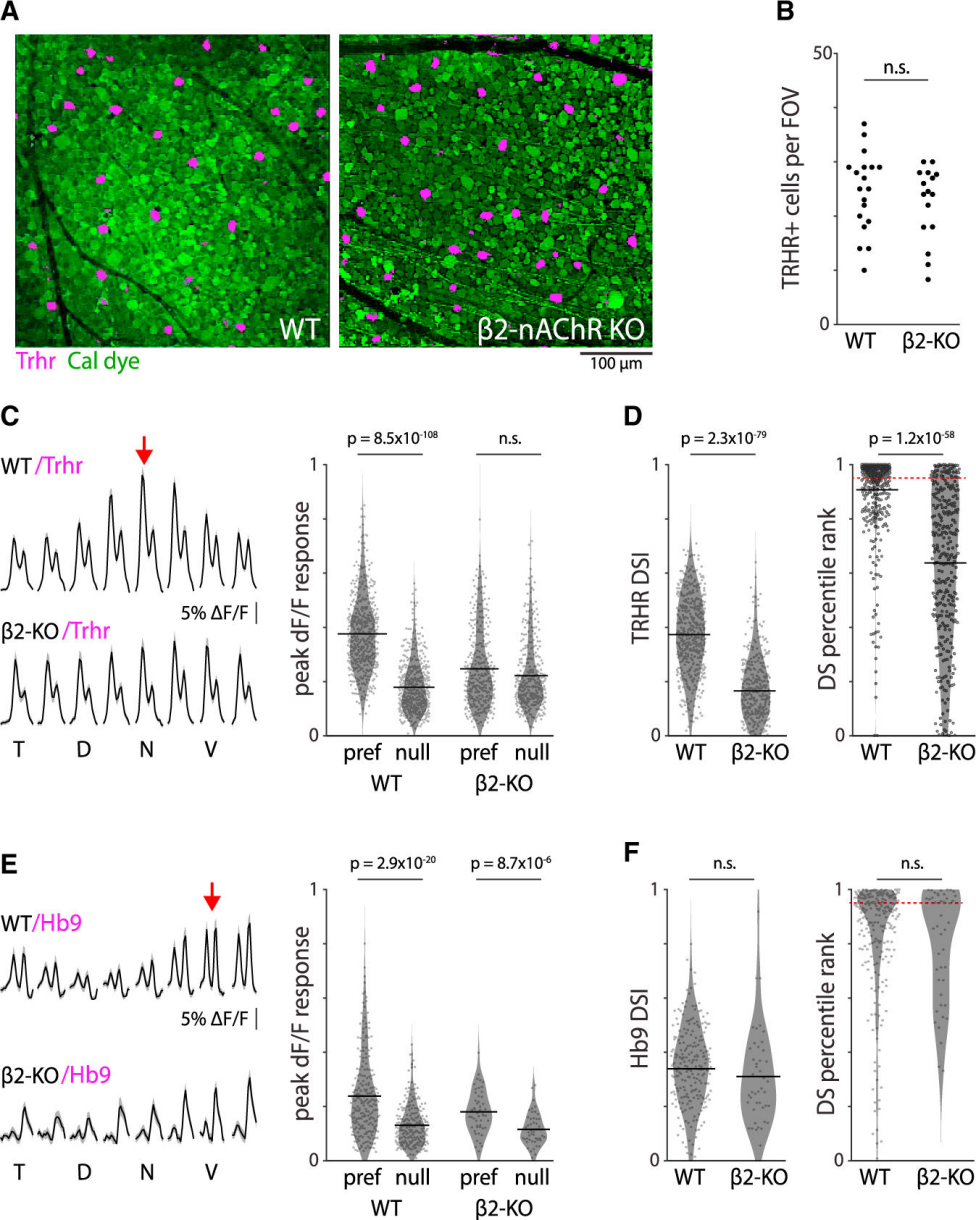
Genetically defined horizontal direction-selective cells in mice that lack cholinergic waves are still present and light sensitive but lose their direction selectivity (A) Two example fields of view (FOVs) showing Trhr-GFP DSGCs (nasal-preferring) in magenta in mice that exhibit normal waves (WT; left) and mice that exhibited reduced cholinergic waves (β2-nAChR-KO; right). The calcium dye Cal-590 AM is depicted in green. (B) Average number of Trhr-GFP DSGCs per FOV in control and β2-nAChR-KO mice. (C) Left: average response of Trhr-GFP DSGCs to bars moving in different directions in both the WT (top) and β2-nAChR-KO (bottom) mice. Shaded area depicts one standard deviation from the mean. Red arrow is the typical preferred direction of Trhr-GFP DSGCs (180° nasal). Right: peak cell response to bars moving in the preferred and null direction in control and β2-nAChR-KO mice. (D) Summary data for DSI (left) and the percentile rank of each cell’s DSI compared with permutations where the directions of the moving bar are block shuffled (right). For reference, 95th percentile is considered statistically significantly DS (red dashed line). N = 465 Trhr-GFP DSGCs across 2 WT mice and 338 Trhr-GFP DSGCs across 2 β2-nAChR-KO mice. (E and F). Same as (C and D) but for Hb9-GFP DSGCs (ventral-preferring). N = 228 Hb9-GFP DSGCs across 4 WT mice and 50 Hb9-GFP DSGCs across 2 β2-nAChR-KO mice. *All statistics here are unpaired t tests. Black horizontal bars denote population mean.

We next assessed the response properties of nasal-preferring (Trhr-GFP) DSGCs in β2-nAChR-KO mice in which a known population of nasal-preferring DSGCs express GFP under the Trhr promoter (Figure 3A). Trhr-GFP cells were present in equal numbers in both WT:Trhr-GFP and β2-nAChR-KO:Trhr-GFP mice (Figure 3B). In β2-nAChR-KO:Trhr-GFP mice, Trhr-GFP DSGCs were equally responsive to drifting bars moving in all directions (Figure 3C) and had significantly lower DSI values compared with the WT:Trhr-GFP mice (Figure 3D, left). Hence, in β2-nAChR-KO:Trhr-GFP mice, many Trhr-GFP DSGCs were not direction selective (Figure 3D, right). To determine if our results were unique to horizontal-preferring DSGCs, we next assessed the response properties of ventral-preferring (Hb9-GFP) DSGCs in β2-nAChR-KO mice in which a known population of ventral-preferring DSGCs express GFP under the Hb9 promoter. Unlike nasal-preferring DSGCs, ventral-preferring DSGCs in β2-nAChR-KO:Hb9-GFP mice exhibited a similar tuning preference to ventral stimuli as in WT:Hb9-GFP mice (Figures 3E and 3F). These data indicate that, in β2-nAChR-KO mice, nasal-preferring DSGCs, and likely most horizontal-preferring DSGCs, respond symmetrically to drifting bars.

**Figure 4.**
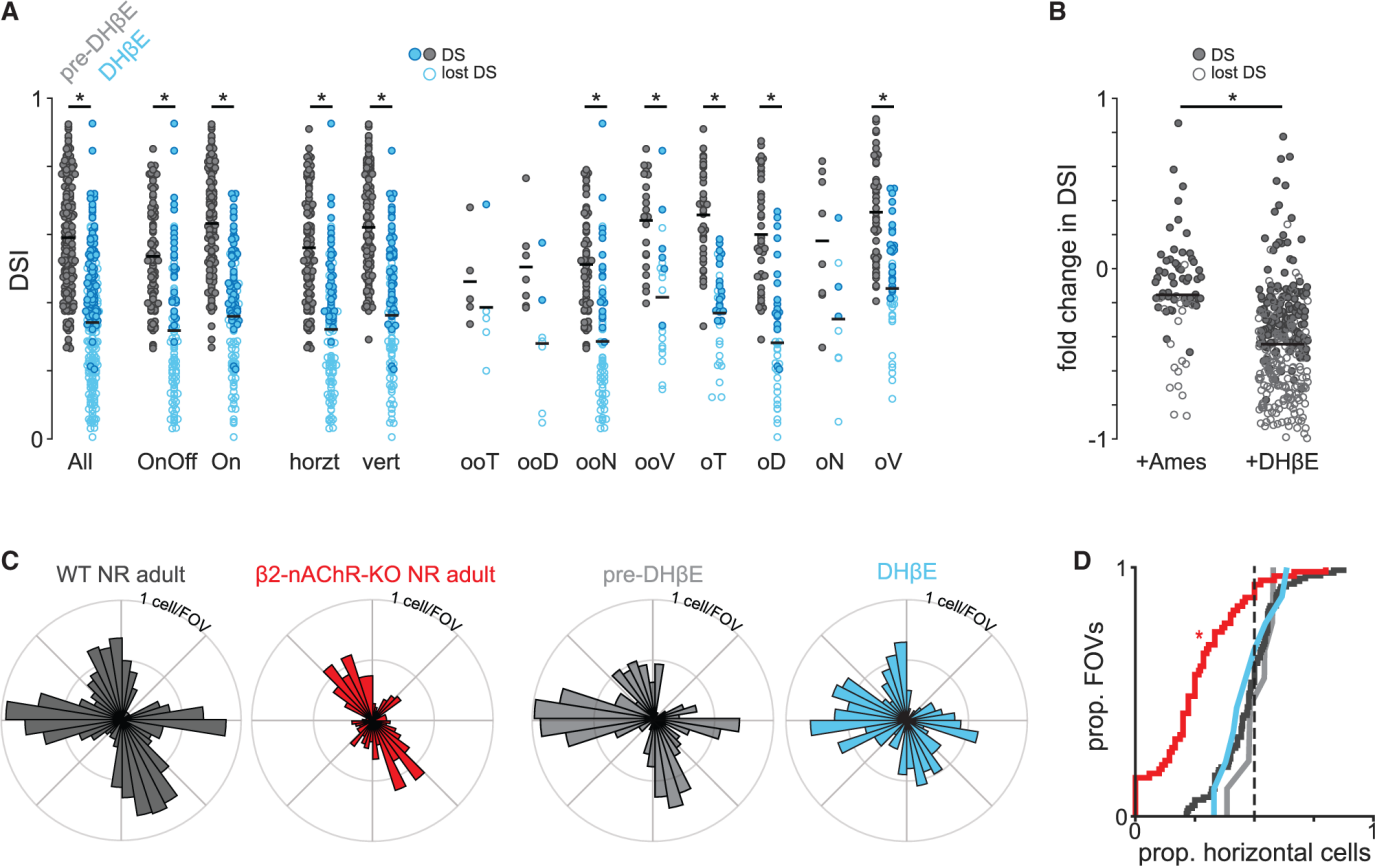
Acute blockade of α4β2- and α4β4-nAChR globally reduces direction selectivity but does not specifically suppress horizontal direction selectivity (A) DSI before (gray) and after (blue) application of a α4β2- and α4β4-nAChR antagonist, DHβE, for all DSGCs among various functional subtypes. Open circles represent cells that lost their direction selectivity according to a permutation test. Horz, horizontal; vert, vertical; oo, On-Off; o, On; T, temporal; D, dorsal; N, nasal; V, ventral. Black horizontal bars represent means. *p < 7.7 × 10−^4^ (paired t test with Bonferroni correction). (B) Fold change in DSI of all DSGCs after the addition of control solution or DHβE. *p < 0.01 (unpaired t test). (C). Direction selectivity (DS) maps of WT (black), β2-nAChR-KO mice (red), WT before addition of DHβE (light gray), and WT after the addition of DHβE (light blue). The black and red plots are repeated from Figure 2 for clarity. (D) Proportions of horizontal-preferring DSGCs per field of view for each of the conditions shown in (C). *Different from WT NR (normal-reared) adult, p < 0.01 (ANOVA followed by Tukey-Kramer post hoc test).

The observed reduction in horizontal direction selectivity in β2-nAChR-KO mice could result from impaired direction-selective circuit development, or directly from reduced signaling through nicotinic acetylcholine receptors. Cholinergic signaling contributes to the direction-selective computation (Sethuramanujam et al., 2016), but whether this contribution is different for horizontal versus vertical direction selectivity is not known. Pharmacological block of nAChRs using dihydro-β-erythroidine (8 μM DHβE), an antagonist targeted to alpha4- and beta2-containing nAChRs (Harvey and Luetje, 1996; Harvey et al., 1996), which potently blocks retinal waves (Bansal et al., 2000), significantly reduced but did not abolish direction selectivity among all DSGCs (Figures 4A and 4B). This reduction in DSI was true for both ON-OFF and ON DSGCs, horizontal- and vertical-preferring DSGCs, and all individual functional DSGC subtypes. Despite the reduction in DSI, the direction selectivity maps in DHβE still included cells that responded to horizontal and vertical motion in proportions similar to pre-DHβE conditions, unlike the direction selectivity map we observed in β2-nAChR-KO mice (Figures 4C and 4D). Therefore, the dramatic and specific loss of horizontal direction selectivity in β2-nAChR-KO mice is not the result of horizontal-preferring DSGCs depending more on β2-nAChRs in response to moving light stimuli than their vertical-preferring counterparts.

**Figure 5.**
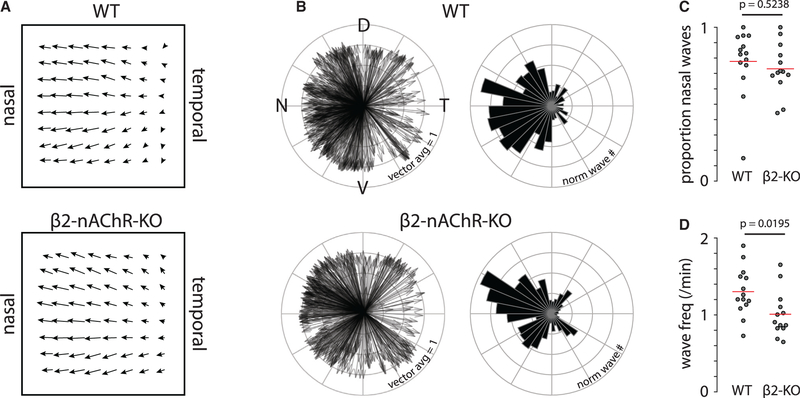
Retinal waves in P9–11 WT and β2-nAChR-KO mice exhibit a propagation bias in the nasal direction (A) Average vector flow of retinal wave propagation in WT (top; N = 834 waves across 14 fields of view in 7 mice) and β2-nAChR-KO (bottom; N = 634 waves across 13 fields of view in 5 mice) retinas. The average vector flow field is obtained from fields of views (850 × 850 μm) imaged in different locations of the retina (ventral, dorsal, nasal, and temporal). (B) Left: polar plot where each arrow represents a retinal wave, the angle represents that wave’s average direction, and the length represents the strength of the directionality (0–1), where 0 means that the wave had no net direction and 1 means that all pixels in the field of view exhibited the same direction). Right: same data represented as polar histograms. (C) Quantification of the proportion of retinal waves that propagate in the nasal direction versus temporal direction. (D) Quantification of the frequency of retinal waves. *All statistics here are unpaired t tests.

It was recently demonstrated that retinal waves preferentially propagate along the axes of forward optic flow at the start of the second postnatal week (P8–P11), and that this propagation bias is critical for setting up direction selectivity in the superior colliculus (Ge et al., 2021). β2-nAChR-KO mice exhibit an 80% reduction in retinal waves in the first postnatal week but exhibit relatively normal frequency of waves in the second postnatal week when the propagation bias is present in WT mice (Burbridge et al., 2014) (Figure 5D). We therefore assessed the propagation bias of retinal waves in P9–P11 β2-nAChR-KO and WT mice using two-photon calcium imaging of large areas of the retina (850 × 850 μm^2^). Both β2-nAChR-KO and WT mice exhibited a pronounced propagation bias in the nasal direction (Figures 5A–5C). Therefore, it is unlikely that the lack of horizontal direction selectivity in β2-nAChR-KO mice is the result of a perturbed horizontal propagation bias.

**Figure 6.**
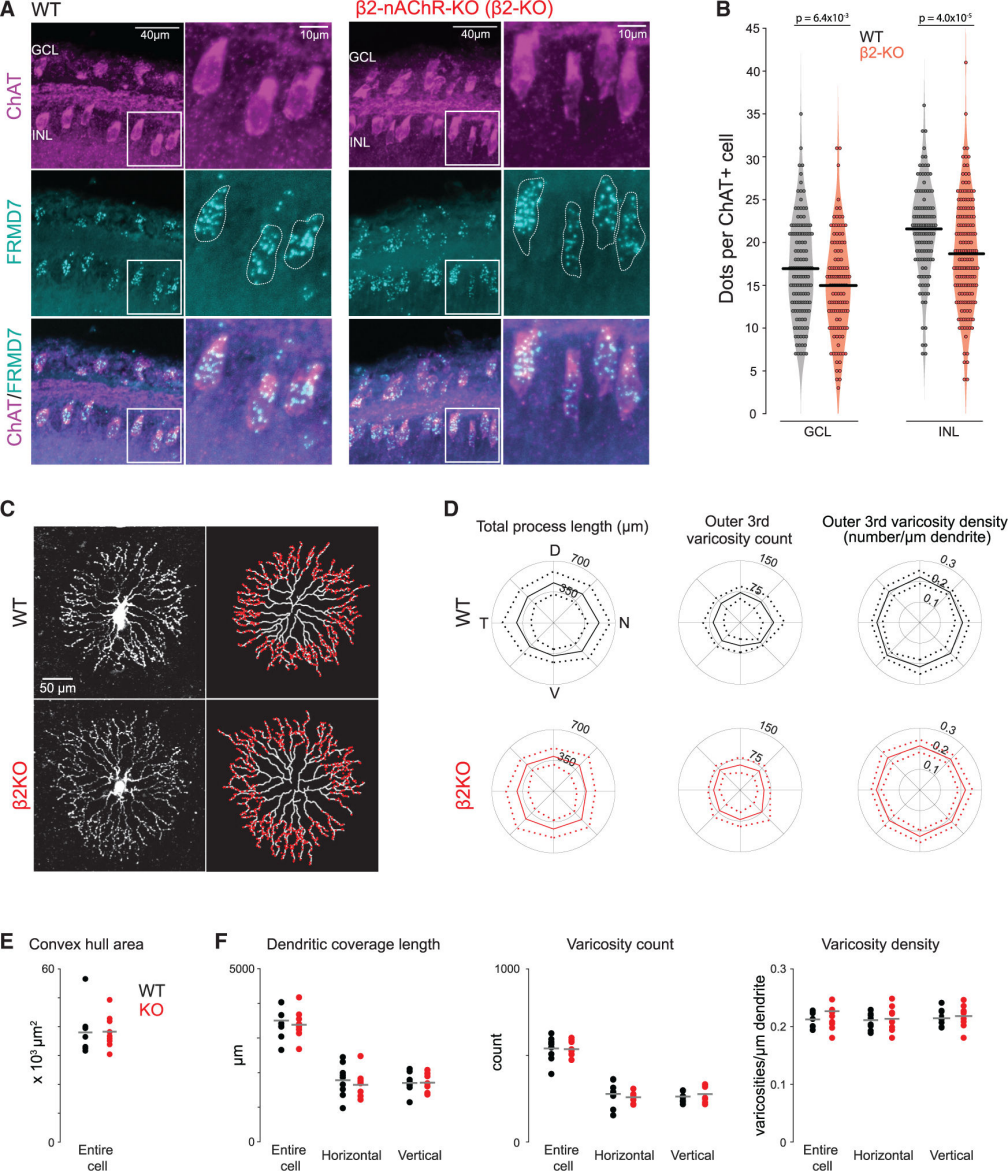
FRMD7 expression and intact starburst amacrine cell morphology persist in mice that lack cholinergic waves (A) Confocal images of WT (left) and β2-nAChR-KO (β2-KO) (right) retinas stained using RNAscope *in situ* hybridization using probes for FRMD7 mRNA (cyan) and immunohistochemistry using ChAT antibody to label starburst amacrine cells (SACs) (magenta). Panels to the right are magnifications of insets in the left panels for each condition. (B) Quantification of FRMD7 mRNA expression counted as punctate dots per ChAT+ cell. WT (black; n = 263 cells in 3 mice) and β2-KO (red; n = 278 cells in 3 mice) retinas in the ganglion cell layer (GCL) (left; WT, n = 136 cells; β2-KO, n = 117 cells) and inner nuclear layer (INL) (right; WT, n = 127 cells; β2-KO, n = 161 cells). Unpaired t test. (C) Maximum-intensity projections of SACs filled with Alexa 488 (left) in WT (top) and β2-KO (bottom). Skeletons were generated from dendritic tracings, and varicosities were counted and overlaid onto traced processes (right). (D) Total dendritic process length (left), varicosity count in outer third of dendritic arbor (middle), and varicosity density in outer third of arbor (right) as a function of directional axis in WT (top, black) and β2-KO (bottom, red). (E) Area of convex hull around SAC dendritic tips. (F) Summary data from left to right: dendritic coverage length, varicosity count, and varicosity density across the whole cell, separated by horizontal and vertical axis. Two-way ANOVA revealed no significant main effect of genotype, direction, or interaction between the two. N = 9 WT cells; 9 KO cells from 3 animals each.

Horizontal direction selectivity is dependent on the FRM domain protein FRMD7, a protein implicated in human nystagmus (Yonehara et al., 2016). FRMD7 mutant mice lack both optokinetic reflex and direction selectivity to horizontal motion, while responses to vertical motion are intact. FRMD7 expression is restricted to starburst amacrine cells (SACs) and emerges during the first 10 postnatal days (Yonehara et al., 2016), coinciding with the occurrence of cholinergic retinal waves, which are greatly reduced in β2-nAChR-KO mice. Using fluorescence *in situ* hybridization combined with immunohistochemistry, we found that, similar to WT littermates, ChAT+ cells in β2-nAChR-KO mice expressed FRMD7 in both the ganglion cell and inner nuclear layers (Figures 6A and 6B), although with a small but significant reduction in expression. These results demonstrate that FRMD7 expression during the first postnatal week persists in the absence of retinal waves. Whether this small reduction in FRMD7 expression is the basis of reduced horizontal direction selectivity in β2-nAChR-KO remains to be determined. Alternatively, the impact of the FRMD7 mutation on the spatiotemporal properties of retinal waves (Ge et al., 2021) may be critical for the development of horizontal direction selectivity.

Finally, we tested whether the loss of horizontal direction selectivity in β2-nAChR-KO mice resulted from perturbation of SAC morphology, which has also been implicated in the establishment of direction selectivity (Kostadinov and Sanes, 2015; Sun et al., 2013). To assess this, we filled SACs in WT and β2-nAChR-KO retinas with a fluorescent dye, acquired two-photon volumetric stacks of filled SACs, and subsequently analyzed SAC dendritic morphology and varicosity distribution relative to each directional axis. We found that SACs in β2-nAChR-KO retinas qualitatively resembled WT SACs in that they were radially symmetric with varicosities located on their distal dendrites (Figures 6C and 6D). Quantification revealed no significant difference in dendritic coverage length or area between SACs in WT and β2-nAChR-KO retinas, even when horizontally and vertically oriented dendrites were considered separately (Figures 6E and 6F). Similarly, varicosity counts and densities in the outer third of the SAC dendritic arbor, where output synapses are located, were unchanged in β2-nAChR-KO.

## DISCUSSION

Here, we report that the spatial organization of preferred directions of DSGCs is mostly established by eye opening and matures normally in the absence of visual experience. Moreover, we make the surprising finding that retinal selectivity to horizontal motion is nearly abolished in β2-nAChR-KO mice, which lack cholinergic retinal waves during the first postnatal week. Using mouse lines in which horizontal DSGCs are labeled, we found that these cells were present and visually responsive in β2-nAChR-KO mice, but their directional tuning was significantly reduced. Together, these findings demonstrate that spontaneous activity prior to vision is critical for the development of horizontal direction selectivity in the retina, while visual experience plays a minimal role. To begin to identify how retinal waves drive the development of direction selectivity, we tested three known mechanisms: propagation bias of retinal waves between P9 and P11 (Ge et al., 2021), expression of FRMD7 mRNA in SACs (Yonehara et al., 2016), and SAC morphology (Kostadinov and Sanes, 2015; Sun et al., 2013). We found that the propagation bias of waves and SAC morphology were indistinguishable between WT and β2-nAChR-KO mouse retinas, and that FRMD7 expression occurred in β2-nAChR-KO, although the levels of expression reached were slightly reduced when compared with age-matched WT mice. Hence, the mechanism by which retinal waves contribute to the establishment of direction selectivity remains a mystery.

*Xenopus* tadpoles, which do not exhibit retinal waves (Demas et al., 2012), begin to experience optic flow early in development as they swim, and this optic flow instructs their retinotopy (Hiramoto and Cline, 2014). It was recently established that the propagation bias of retinal waves in mice might provide a similar instructive cue to developing direction-selective circuits (Ge et al., 2021). This study revealed that retinal waves mimic the type of optic flow that animals will eventually experience as they navigate their environment, and that perturbing the propagation bias of waves perturbs collicular direction selectivity. Therefore, it is possible that retinal waves confer directional information onto developing retinal circuits before conventional photoreceptors come on line, and in so doing contribute to the establishment of visual maps before mice have cone- and rod-mediated visual experience. However, the horizontal propagation bias of retinal waves in β2-nAChR-KO mice was intact, suggesting that another wave-related mechanism may be involved in the development of direction selectivity.

The lack of horizontal direction selectivity in β2-nAChR-KO mice is strikingly similar to the phenotype exhibited by FRMD7 mutants. In this study, we show that FRMD7 expression in β2-nAChR-KO mice develops to almost normal levels by the end of the first postnatal week. Therefore, it is unlikely that this small reduction in FRMD7 expression compared with WT mice explains the near total lack of horizontal direction selectivity in β2-nAChR-KO mice, though this remains to be tested. One possibility is that FRMD7 and retinal waves independently influence different critical components of the horizontal DS circuit (Hamilton et al., 2021). Alternatively, a recent study found that FRMD7 mutant mice lack the nasal propagation bias that is exhibited in P8–P11 WT mice (Ge et al., 2021). These findings suggest that FRMD7 might somehow set the scaffold that will mediate the propagation bias of waves, which in turn establishes direction selectivity in the superior colliculus. Ultimately, however, whether the mechanisms implicated in establishing direction selectivity in the superior colliculus are the same as those in the retina remains to be determined.

These findings indicate that different mechanisms underlie the development of horizontal versus vertical direction selectivity. What mediates the development of the vertical map? One possibility is that the asymmetric inhibitory circuits that mediate vertical direction selectivity mature later, potentially depending on late-stage glutamatergic waves, which are spared in β2-nAChR-KO mice. Alternatively, the distribution of preferred directions along the vertical axis may rely on molecular gradients, such as the EphB and their ephrin ligands, which are expressed transiently in the retina during the developmental period when direction-selective circuits develop (Thakar et al., 2011).

### Limitations of the study

One limitation of our study is that our recordings were confined to the ventral retina, where we were able to stimulate UV cones without compromising our two-photon calcium imaging. Hence, we were able to obtain a complete map near the vertical singularity, which resides in the ventral retina, but not near the horizontal singularity, which resides in the temporal retina. This may explain why we were able to capture the change in preferred directions as a function of retinal location in vertical-selective DSGCs but not horizontal-selective DSGCs (Figure 1E).

Although we tested various hypotheses (propagation bias, FRMD7 expression, SAC morphology) to explain the loss of horizontal direction selectivity in β2-nAChR-KO mice, we have not yet identified the underlying mechanism. Acute blockade of some nAChRs with DHβE experiments did not recapitulate the β2-nAChR-KO phenotype but did lead to an overall reduction of direction selectivity. Given this, we are left to hypothesize that either cholinergic waves or chronic activity via β2-nAChRs during development play a role in setting up horizontal direction selectivity. Both hypotheses can be resolved by reversibly abolishing cholinergic waves, but the currently available methods (multiple intraocular injections) must be repeated too often to accomplish a complete block of waves over 4 days (estimated by Ge et al. [2021] to last 6 h) and greatly deteriorate the integrity of the retina, making recordings difficult.

## STAR★METHODS

### RESOURCE AVAILABILITY

#### Lead contact

All correspondance should be directed to Marla B. Feller (mfeller@berkeley.edu).

#### Materials availability

Blueprint for building the custom-made light meter can be found here: https://github.com/Llamero/Light_Color_and_Intensity_Datalogger.

#### Data and code availability

- All data reported in this paper will be shared by the lead contact upon request.
- All original code has been deposited at https://github.com/FellerLabCodeShare/DS-Map-Project and is publicly available as of the date of publication. DOIs are listed in the key resources table.
- Any additional information required to reanalyze the data reported in this paper is available from the lead contact upon request.

### EXPERIMENTAL MODEL AND SUBJECT DETAILS

#### Animals

All animal procedures were approved by the UC Berkeley Institutional Animal Care and Use Committee and conformed to the NIH *Guide for the Care and Use of Laboratory Animals*, the Public Health Service Policy, and the SFN Policy on the Use of Animals in Neuroscience Research. All mice used in this study were p9–60 and of both sexes. For the majority of experiments, we used C57B6 mice. In a subset of experiments, we used transgenic mice where Hb9 cells (ventral-preferring DSGCs) or Trhr or Drd4 cells (nasal-preferring DSGCs) were tagged with a GFP marker. These transgenics were on the C57B6 genetic background. To study the role of spontaneous activity, we used β2-nAChR-KO mice where the beta subunit of the nicotinic acetylcholine receptor is knocked out. To study the role of spontaneous activity on known cell types, we bred β2-nAChR-KO mice with either Hb9-GFP or Trhr-GFP transgenic mice. To study starburst amacrine cell morphology, we crossed Chat-Cre with nGFP mice.

To study the effects of visual deprivation on the development of direction selectivity maps, mice were born and raised in rooms that either had 12-h day/night cycle (NR and EO groups) or 24 darkness (DR group). For the dark-reared mice, all animal husbandry was conducted with red light, which minimizes stimulation of photoreceptors. Deprivation of light in the dark-reared mice was confirmed using a custom-built light meter attached to cages that logged data at 1Hz over the course of a month (https://github.com/Llamero/Light_Color_and_Intensity_Datalogger).

### METHOD DETAILS

#### Retina preparation

Mice were deeply anesthetized with isoflurane inhalation and euthanized by decapitation. Eyes were immediately enucleated and retinas were dissected in oxygenated (95% O_2_/5% CO_2_) Ames’ media (Sigma) at room temperature under infra-red illumination. Cuts along the choroid fissure were made prior to isolating the retina from the retinal pigmented epithelium. These cuts were made to precisely orient retinas and reduce orientation variability between preparations (Stabio et al., 2018). Isolated retinas were mounted whole over white filter paper (Whatman) with the photoreceptor layer side down, and transferred in a recording chamber of an upright microscope for calcium dye loading and subsequent imaging. The whole-mount retinas were continuously perfused (3 mL/min) with oxygenated Ames’ media at 32 to 34°C for the duration of the experiment. Retinas were bolus loaded with either the green calcium dye Cal 520 AM or the red calcium dye Cal 590 AM. The retina from the other eye was kept in the dark at room temperature in Ames’ media bubbled with 95% O_2_, 5% CO_2_ until use (maximum 8 h).

#### Two-photon calcium imaging

Two-photon fluorescence measurements were obtained with a modified movable objective microscope (MOM) (Sutter instruments, Novator, CA) and made using a Nikon 16X, 0.80 NA, N163LWD-PF objective (Nikon, Tokyo, Japan). Two-photon excitation of calcium dyes was evoked with an ultrafast pulsed laser (Chameleon Ultra II; Coherent, Santa Clara, CA) tuned to 920 nm for green dyes and GFP or 1040 nm for red dyes. The microscope system was controlled by ScanImage software (www.scanimage.org). Scan parameters were (pixels/line x lines/frame [frame rate in Hz]): (256 × 256 [2.96Hz]), at 1 ms/line. This MOM was equipped with a through-the-objective light stimulation and two detection channels for fluorescence imaging.

A previous study reported that a 2-photon laser at appropriate power for imaging (5–10 mW) can induce light responses (Euler et al., 2009). To account for 2-photon-mediated responses, we adapt retinas to the laser with a 5-min imaging session, a strategy outlined in the original paper.

To keep track of the location of the retina, we zeroed the micromanipulator that moves the objective on the optic nerve, and oriented ventronasal retina along the x axis, and ventrotemporal along the y axis (or vice versa). For every field of view, we kept track of the x and y distance from the optic nerve. At the end of the experiment, we replaced the objective with a 10X objective and collected bright field images of the whole retina. We used this image of the whole retina to determine how radially offset ventronasal and ventrotemporal retina were from the x and y axis. Across all of our preparation, ventronasal and ventrotemporal retina were offset from the x and y axis by an average of −4.9°C with a standard deviation of 11.9°C.

The same microscope was used to image retinal waves but the field of view was set to 850um × 850um and the scan parameters were [pixels/line x lines/frame (frame rate in Hz)]: [128 × 128 (5.92 Hz)], at 1 ms/line. The same field of view was imaged until at least 50 retinal waves had propagated through it.

#### Interline visual stimulation

Visual stimuli patterns were generated in matlab using the psychphysics toolbox and projected onto the retina using a digital micromirror device that contains an LED (UV: 375 nm). To decrease the background signal entering the photomultiplier tubes due to UV stimulation of the calcium dye, the stimuli was delivered on the flyback of the fast axis scanning mirror during a unidirectional scan so as to interleave the stimuli with the imaging. The rate at which the visual stimulus was shown with the interline (1 KHz) is faster than the flicker fusion frequency for mice (approximately 15–20 Hz) (Tanimoto et al., 2009). The intensity of the UV stimulus was 2 × 10^6^ photons s^−1^ um^−2^. Our stimulus was a rectangle (width = 500 μm, length = 1000 μm) that drifted across the field of view in 8 different directions (every 45°C) at speed of 250 um/s, which is ideal for activating both On and On-Off DSGCs (Dhande et al., 2013; Oyster, 1968). Each direction was repeated 3 times in a block shuffled manner so that all 8 directions were presented before repeating, with 10 s of downtime between stimuli. In all cases, the stimuli were shown on a dark background (intensity of dark background = 2 × 10^3^ photons s^−1^ um^−2^).

The same MATLAB code that generated the visual stimuli also generated a text file that contained metadata for every trial, like the width/length/speed of the bar, the direction the bar was moving in during each trial, and the start and end time of each trial.

#### Pharmacological experiments

We blocked the α_4_β_2_ and the α_4_β_4_ subunits of the nicotinic acetylcholine receptors via the application of DHβE (8 μM; Tocris, part 2349).

#### Fluorescent *in situ* hybridization and immunohistochemistry

Fluorescent *in situ* hybridization (FISH) on mice retinal sections was carried out using the RNAscope Multiplex Fluorescent Assay (Advanced Cell Diagnostics) according to the manufacturer’s instructions. Briefly, dissected retinas from P12 wild type and β2-nAChR-KO littermates were fixed with 4% paraformaldehyde, cryoprotected with 30% sucrose in PBS, frozen on dry ice, and sectioned on a microtome at 20 μm. Then, sections were hybridized with probes for mouse FRMD7 mRNA (ACD bio; 432,391), signals were amplified, and fluorescently tagged with Opal-620 (PerkinElmer, FP1495A). To label starburst amacrine cells, we used immunohistochemistry using ChAT antibody (Millipore; AB144P). Briefly, after FISH was completed, sections were rinsed and blocked in 10% normal donkey serum TBS-1% BSA blocking solution, incubated in primary antibody (Goat-anti-ChAT; 1:200), incubated in secondary antibody (Anti-goat Alexa 568; 1:1000), with rinses in between, then mounted with Prolong Gold (Life Technologies).

#### Starburst amacrine cell morphology

We used whole mount retinas in the ChAT:nGFP/β2-nAChR-KO mouse line to target and fill starburst amacrine cells (SACs) with fluorescent dye under 2-photon illumination at 920nm (imaging as previously described in [Morrie and Feller, 2018]). Mice used were ~P30 littermates that were heterozygous or homozygous for the β2-nAChR-KO allele. To fill SACs, we approached nGFP-expressing ON-SACs with bent-tip sharp electrodes filled with 2mM Alexa-488, and once inside the cell we applied a −20nA current for 500ms. This led to complete filling of the cell body, dendritic arbor, and varicosities. Z-stacks were taken at 512 × 512 pixel resolution, 4x digital zoom, and 4 frames per Z section.

### QUANTIFICATION AND STATISTICAL ANALYSIS

#### Statistical tests

Power analyses were computed using G-Power (Faul et al., 2007) and statistical tests were computed in MATLAB. Details of statistical tests, number of samples, and p values are listed in the figure captions. Unless otherwise stated, p < 0.01 was considered significant.

#### Image processing

Raw movies were motion-corrected and normalized into ΔF/F_0_ automatically using a custom-made FIJI macro that was run in ImageJ v1.52n (macro name in project’s GitHub folder: RegisterAndCalcDFOF_withGFP_v2.ijm). Briefly, 1) movies were motion corrected using the “correct 3D drift” plugin in FIJI on a duplicate of the raw data that had been averaged in the time dimension (zMean = 30 s) – Note that this step can only correct xy drift, z drift cannot be corrected and hence was prevented during acquisition. 2) Frames where the light stim occurred were removed to isolate baseline F. 3) the baseline F was subtracted from the raw F movie, and this result was divided by the baseline F. The resulting ΔF/F_0_ movies were then transferred to MATLAB for further image analysis.

#### Semi-automatic detection of DSGCs

A MATLAB code was written to automatically identify potential ROIs within a ΔF/F_0_ movie that were direction selective. Briefly, the code analyzes every pixel in the x-y dimension by 1) taking a mean average of its neighbor pixels, 2) computing the average peak ΔF/F_0_ response for every stimulus direction and 3) computing the vector sum (VS) and direction selectivity index (DSI) for each pixel.

$$Vector\;sum\;=\frac{\sum_{n=1}^{8\;\text{directions}}\left(xn,\;yn\right)}{\sum_{n=1}^{8\;\text{directions}}mean(peak\;(\Delta F/F0n))}$$

where x_n_ and y_n_ are the cartesian coordinates of the polar vector where the direction of the vector is the stimulus direction and the length of the vector is the average peak ΔF/F_0_ for that direction. The direction of the vector sum is the ROI’s preferred direction, which is used to calculate DSI, and the length of the vector sum is the magnitude of the tuning.

Directionselectivit/yindex=(ΔF/F0pref−ΔF/F0null)/(ΔF/F0pref+ΔF/F0null)

where pref is the direction angle closest to the vector sum’s direction and null is 180°C rotated from pref.

Next the MATLAB code used a 2D median filter to enrich the cell-like ROIs that exhibit similar preferred directions. The resulting image is then overlaid on an average fluorescence image of the motion-corrected movie and oval ROIs are drawn in FIJI over regions that were mathematically determined to be DS and that also correspond to an anatomical cell. These ROIs are then transferred to MATLAB for further analysis.

#### Manual classification of On-Off and On cells

A MATLAB code was written to present a user with a GUI that cycled between every identified cells. The GUI was customized to display the chronological ΔF/F_0_ trace of the cell, the 3 blocked and averaged ΔF/F_0_ traces for each of the eight different directions, and a tuning plot of the cell using the blocked traces. Using this GUI, a user classified cells as either “On-Off” if they exhibited a ΔF/F_0_ peak at the onset and offset of the moving bars, “On” if they exhibited a ΔF/F_0_ peak only at the onset of the moving bars, and “Bad” if the cells exhibited grossly inconsistent responses to each of the 3 trials for the eight different directions.

#### Statistical determination of direction selectivity

The following statistical approach was used to determine which cells were significantly direction selective: For each cell, the DSI was first calculated. Then, for 1000 permutations *in silico*, the directions of the moving bar stimuli were block-shuffled and the DSI was again calculated. The cell’s DSI calculated from the non-permuted dataset was ranked against all of the DSIs calculated from the permuted dataset. If the cell’s actual DSI ranked higher than 95% of the permuted DSIs, it was determined to be significantly direction selective.

#### Clustering analysis

We used the same clustering method that was described in a previous study (Bos et al., 2016). Briefly, K-means clustering analysis in MATLAB software was used to evaluate the pattern of distribution of the preferred directions from both On-Off and On DSGCs. Note that because the preferred directions of On DSGCs in our dataset seemed to follow the same directions as their On-Off counterparts, similar to what was described previously (Sabbah et al., 2017), we ultimately combined On-Off and On groups when performing the clustering. All the lengths of the preferred directions were fixed to 1 and these were transformed into Cartesian coordinates for subsequent angular distance measurement. This method optimizes the set of clusters with respect to the distance between each point and the centroid of its cluster, summed for all points. We compared 2–8 cluster numbers, and we calculated the fitness of clustering by using the silhouette value (SV).

SV(i)=(b(i)−a(i))/max(a(i),b(i))

where a(i) is the average distance between I and all other data within the same cluster (called measure of cohesion), and b(i) is the average distance between I and all points in the nearest cluster (called measure of separation from the closest other cluster). A SV close to 1 indicates data perfectly clustered, whereas a SV close to 0 reflects data which are ambiguously clustered.

Since a subset of experiments were performed in transgenic mice where known DSGCs were labelled with GFP, we used these known cell types to define the clusters. For example, the cluster that pointed ventrally and matched the Hb9-GFP, which labels a subset of ventral-preferring cells, was defined to be the ventral cluster of DSGCs. Immediately clockwise of this cluster, the cluster that pointed nasally and matched the Drd4-GFP, which labels a subset of nasal-preferring cells, was defined to be the nasal cluster. The cluster 180° C rotated from the defined ventral cluster was defined as the dorsal cluster, and the cluster 180°C rotated from the nasal cluster was defined as the temporal cluster. Because the preferred directions change as a function of retinal location this analysis was performed separately for ventronasal and ventrotemporal retina, as well as for central (<1000 μm from optic nerve) and peripheral fields of view (≥1000 μm from optic nerve).

#### Quantitative properties of the direction selectivity map

From the dataset that is classified as On-Off and On cells and for each directional cluster, we computed the proportion of cells, the DSI, the VS, and the variance of angles within each class of DSGCs. Cumulative distribution plots were used to present the data. Differences between groups was determined by performing an ANOVA followed by a Tukey-Kramer post-hoc test to account for unequal sample sizes.

#### Analysis of FRMD7 expression

Z-stacks of each retina were acquired using confocal microscopy (CRL Molecular Imaging Center, Berkeley, CA) at 1040 × 1040 pixel resolution, 40x digital zoom, and 1 μm per Z section to obtain a total Z volume of 10–15 μm. Positive FRMD7 expression was identified as punctate dots present within the cells that are ChAT + via fluorescent antibody staining, where FRMD7 expression is restricted in wildtype mice (Yonehara et al., 2016). We manually counted dots per SAC in both ganglion cell and inner nuclear layers using the ROI multipoint tool in ImageJ.

#### Analysis of SAC morphology

Z-stacks were processed by applying a 2-frame median 3D filter and then averaging 4 frames per Z-section to reduce noise. SAC processes were subsequently traced in 3D using the FIJI/ImageJ Simple Neurite Tracer (SNT) plugin (Arshadi et al., 2021). Varicosities were counted and associated with processes using multipoint tool within SNT. A custom MATLAB script was used to quantify morphological features such as length and number of dendritic processes and varicosities, their angle relative to the soma and the ventral direction, and the convex hull area of SAC dendritic arbors. Analysis of varicosities was limited to the outer third of the dendritic arbor, as this is where GABAergic output synapses are located (Ding et al., 2016; Vlasits et al., 2016).

#### Analysis of wave propagation

Retinal wave movies were processed using the following pipeline. First, a dF was calculated by using a moving average of the baseline fluorescence that did not include wave events. Next, the signal was enriched by applying a 3 d Gaussian filter with settings of 2 × 2 × 2 pixels. A custom-built FIJI macro was then used to identify individual wave events and calculate the direction of propagation at various regions in the field of view (https://github.com/Llamero/Retinal_Wave_Vector_Flow-Macro; use draft 16). The resulting waves and their vector flow fields were then transferred to MATLAB for plotting and quantification of propagation bias and frequency. For the quantification of propagation bias, any wave with a + x vector was considered to be propagating temporally and any wave with −x vector was considered be propagating nasally.

## Supplementary Material

1

## Figures and Tables

**KEY RESOURCES TABLE:** 

REAGENT or RESOURCE	SOURCE	IDENTIFIER
Antibodies		
Goat polyclonal anti-Choline Acetyltransferase (ChAT)	Millipore	CAT# AB 144P; RRID: AB_2079751
Donkey anti-goat Alexa Flour 568	Invitrogen	CAT# A-11057 RRID: AB_2534104
Chemicals, peptides, and recombinant proteins		
Dihydro-β-erythroidine hydrobromide	Tocris	CAT# 2309
CalBryte 590 AM	AAT Bioquest	CAT# 20700
CalBryte 520 AM	AAT Bioquest	CAT# 20650
Ames' Media	Sigma	Cat# A1420-10X1L
Experimental models: Organisms/strains		
Mouse: B2-nAChR-KO	(Bansal et al., 2000)	N/A
Mouse: B2-nAChR-KO:Trhr-GFP	Bred in lab	N/A
Mouse: Trhr-GFP	(Rivlin-Etzion et al., 2011)	N/A
Mouse: B2-nAChR-KO:Hb9-GFP	Bred in lab	N/A
Mouse: B6.Cg-Tg(Hlxb9-GFP)1Tmj/J	The Jackson Laboratory	005029
Mouse: Drd4-GFP	(Rivlin-Etzion et al., 2011)	N/A
Mouse: C57BL/6J	The Jackson Laboratory	000664
Mouse: B6.129-*Gt(ROSA)26Sor^tm1Joe^*/J (nGFP)	The Jackson Laboratory	008606
Mouse: B6;129S6-*Chat^tm2(cre)Lowl^*/J (ChatCre)	The Jackson Laboratory	006410
Mouse: B2-nAChR-KO:ChatCre:nGFP	Bred in lab	N/A
Software and algorithms		
MATLAB	Mathworks	https://www.mathworks.com/products/matlab.html; RRID: SCR_001622
ScanImage	Vidrio Technologies	http://scanimage.vidriotechnologies.com/display/SIH/ScanImage+Home; RRID: SCR_014307
FIJI	NIH	https://imagej.nih.gov/ij; RRID:SCR_003070
Simple Neurite Tracer FIJI plugin	NIH	https://imagej.net/SNT
G*Power	HHU	https://www.psychologie.hhu.de/arbeitsgruppen/allgemeine-psychologie-und-arbeitspsychologie/gpower
Custom-made analysis code	This paper	https://github.com/FellerLabCodeShare/DS-Map-Project
Other		
RNAscope Probe Mm-Frmd7	Advanced Cell Diagnostics	CAT# 432391
Opal-620	Perkin Elmer	FP1495A

## Highlights

- Visual experience plays minimal role in maturation of direction selectivity map
- Horizontal direction selectivity is dramatically reduced in β2-nAChR-KO mice
- The propagation bias of retinal waves in β2-nAChR-KO mice is similar to WT
- β2-nAChR-KO retinas have normal SAC morphology and near-normal levels of FRMD7
