# Supplementary material for: The influence of spontaneous and visual activity on the development of direction selectivity maps in mouse retina
Source: Cell Rep. Author manuscript; Available in PMC 2022 Feb 1. (PMC8805704; doi:10.1016/j.celrep.2021.110225)
Supplement: 1 [file NIHMS1771088-supplement-1.pdf]

Cell Reports, Volume *38*

Supplemental information

The influence of spontaneous and visual activity on the development of direction selectivity maps in mouse retina

Alexandre Tiriac, Karina Bistrong, Miah N. Pitcher, Joshua M. Tworig, and Marla B. Feller

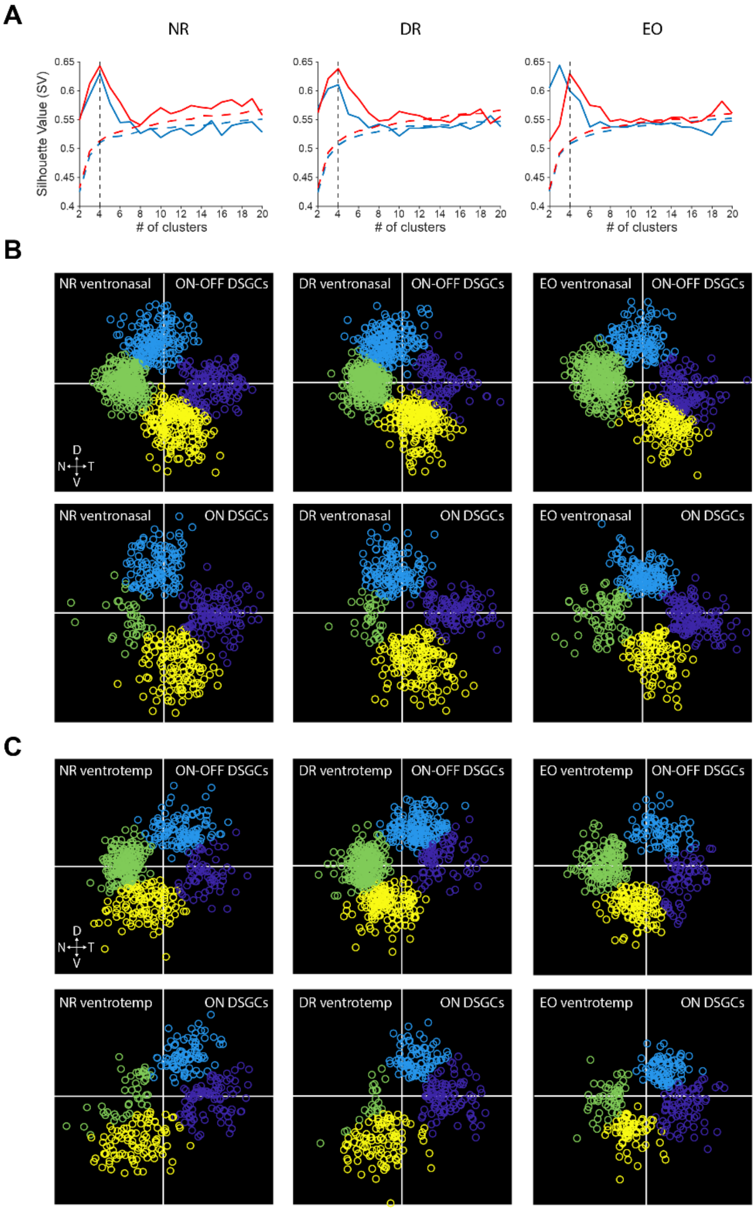

Figure S1. Method used to functionally cluster DSGCs, related to Figure 1.
A. Silhouette analysis for ON-OFF (blue) and ON (red) DSGCs for the ventronasal quadrant of normal-reared (NR), dark-reared (DR), and eye opening (EO) mice. The silhouette value is a measure of how segregated the clusters are, with higher values signifying higher segregation. The red and blue dotted lines are the average of simulated data where the preferred directions were randomized. The black dotted lines indicate a cluster number of 4.
B. Results of functionally-clustering ventronasal data into 4 groups, which are defined as temporal, nasal, dorsal, and ventral clusters. For each data point, the angle is the cell's preferred direction, and the length from origin is its vector sum.
C. Same as B but for ventrotemporal data.

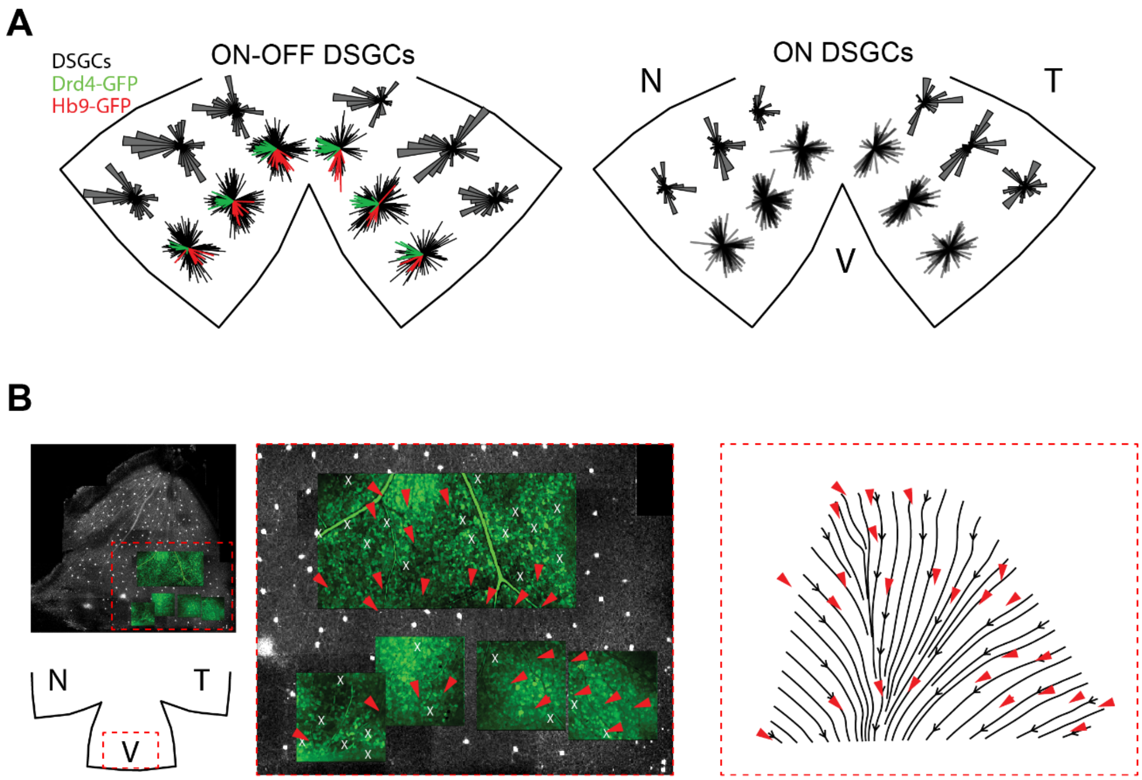

Figure S2. Vertical-preferring DSGCs point toward the ventral pole, related to Figure 1
A. Polar and rose plots depicting the local direction selectivity map at various locations of the retina. Green and red lines depict the preferred directions of genetically-identified subtypes of nasal-preferring (Drd4) and ventral-preferring (Hb9) DSGCs.
B. Maps of the preferred direction of ventral-preferring DSGCs (Hb9-GFP) surrounding the ventral pole. Left panels depict imaging FOV. Middle panel is a zoom in of left, with the red arrows showing the preferred directions of Hb9-GFP DSGCs. White X depict unresponsive Hb9s. Grey image in the background depicts non-imaged areas of the retina. Right panel is a vector flow map generated in MATLAB based on recorded preferred directions of the Hb9-GFP DSGCs at left. Vector flow appears to converge on ventral pole.

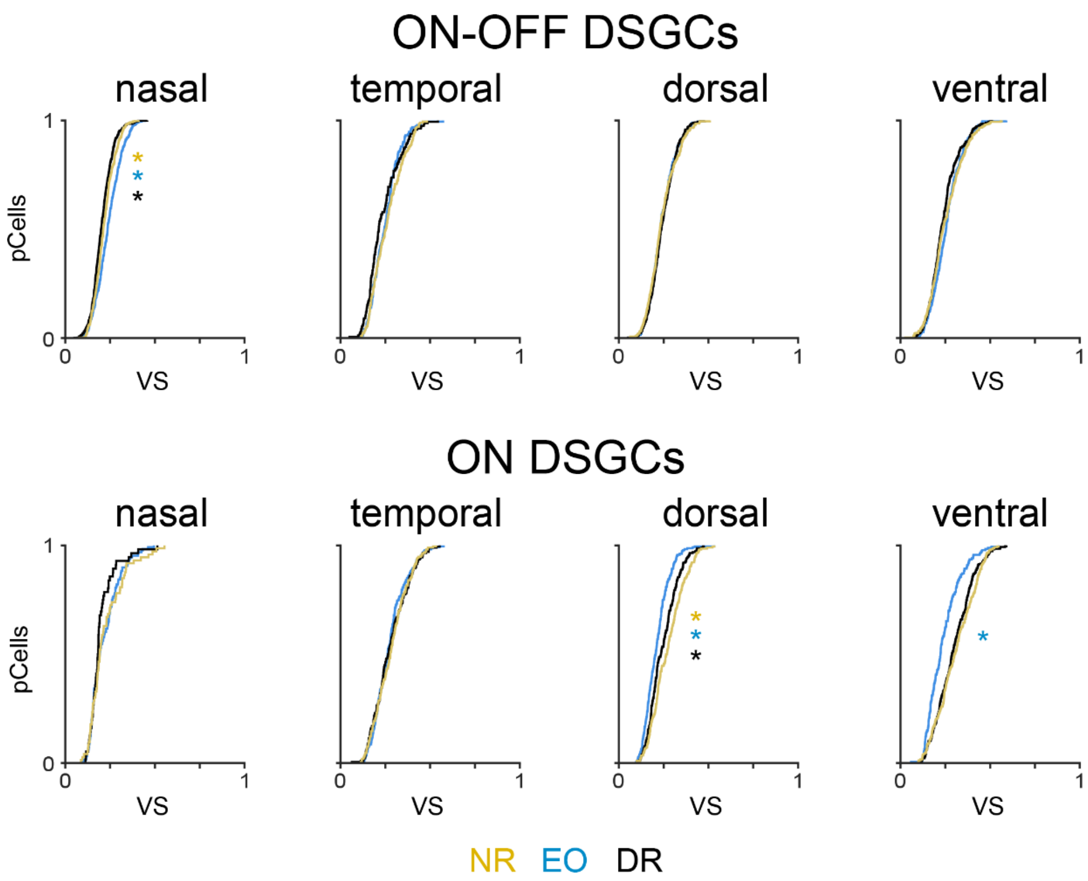

Figure S3. Summary results for directional tuning using vector sum, related to Figure 1.
Cumulative distribution plots for vectors sum (VS) values for each DSGC subtype in retinas isolated from normally-reared mice (NR), at eye opening (EO) and dark-reared mice (DR). Vector sum is a complementary measure to direction selectivity index (DSI) of directional tuning. The significant differences in the vector sum results mirror what we observed in the DSI results. * different from other experimental group, $p < 0.01$.

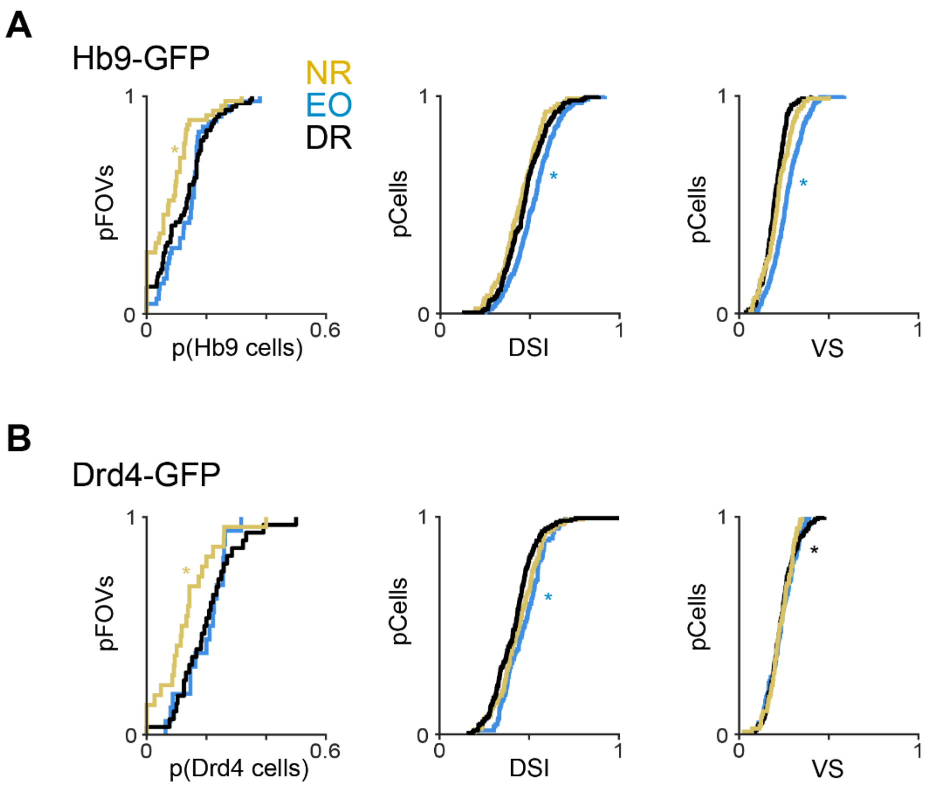

Figure S4. Summary results for the genetically identified subset of vDSGCs (Hb9-GFP) and nDSGCs (Drd4-GFP), related to Figure 1.

A. Left: Cumulative distribution plots depicting the proportion of field of views that exhibit different proportions of Hb9-GFP DSGCs in retinas isolated from normally-reared mice (NR), at eye opening (EO) and dark-reared mice (DR). Middle and Right: Cumulative distribution plots for direction selectivity tuning computed as both direction selectivity index (DSI, middle) and vector sum (VS, right). FOV, field of view. * different from other experimental group, $p < 0.01$.

B. Same as A but for Drd4-GFP DSGCs.

In contrast to total population of DSGCs (Figure 1F), we observe a slightly but significantly lower proportion Hb9-GFP and Drd4-GFP DSGCs in normally-reared adult mice. With respect to tuning strength, Hb9-GFP and Drd4-GFP DSGCs are slightly but significantly more tuned at eye opening than in either adult group. There are no differences in tuning strength between normal- and dark-reared adults.

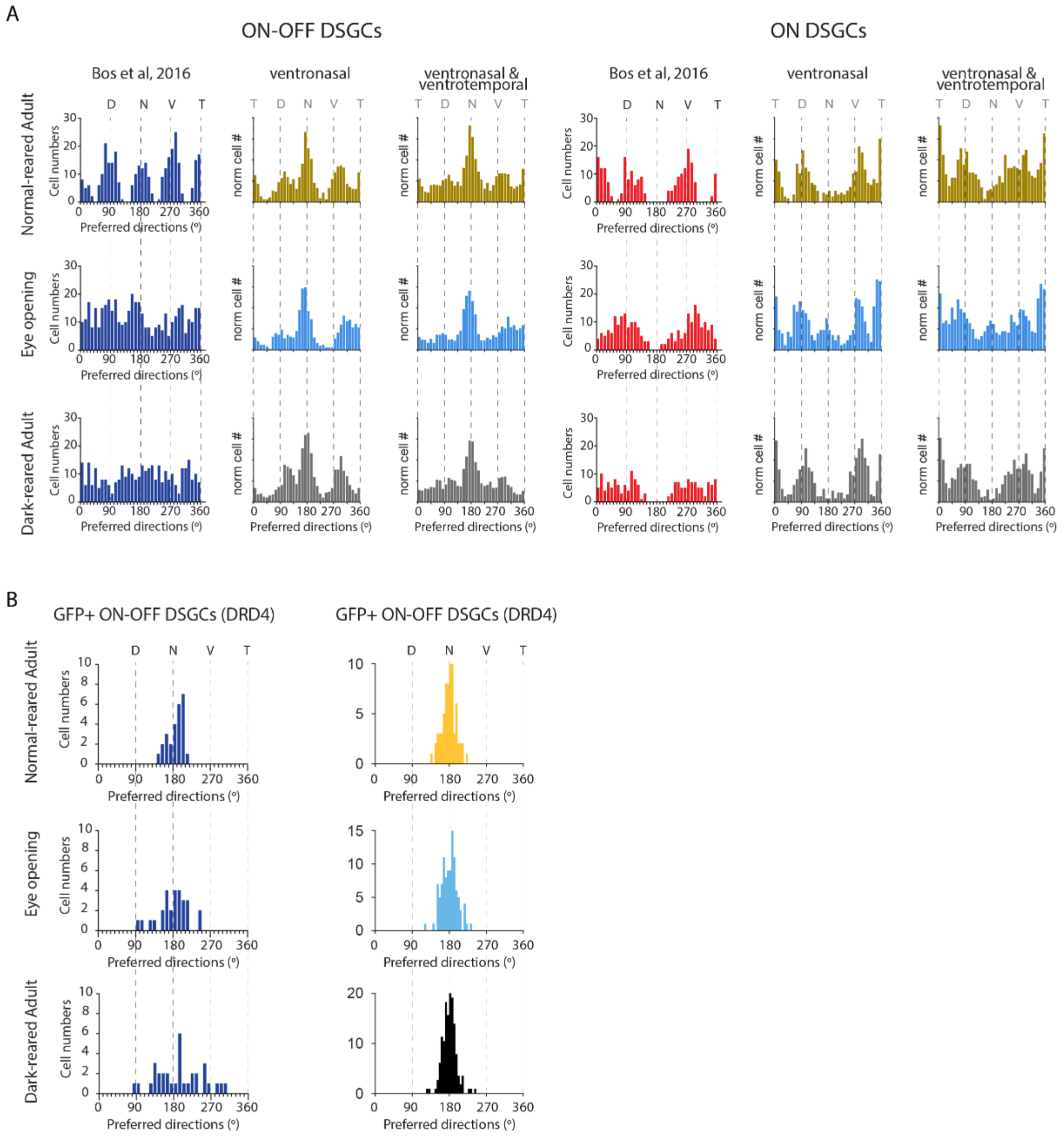

Figure S5. Direct comparison of distributions of preferred directions of DSGCs of this study with previous study, related to Figure 1.

A. Left: Distribution of preferred directions from Bos et al 2016 in which we did not keep track of nasal vs. temporal location of imaging field of views (FOVs). Middle: Data from current study for FOVs in ventronasal quadrant. Right: Data from current study in which data from ventronasal and ventrotemporal are combined. Data are shown for both ON-OFF and ON DSGCs in normally-reared mice (top), at eye opening (middle) and dark-reared mice (bottom). Right: Same as A for ON-DSGCs

B. Distribution of preferred directions of subset of nasal-preferring DSGCs (Drd4-GFP) from Bos et al 2016 (left) and this current study (middle).

Discussion: In (**Bos et al, Current Biology 2016)**, we reported that ON and ON-OFF DSGCs were directionally tuned at eye opening in mice, but that their preferred directions are not clustered along the cardinal axes as in adults. We also reported that this diffuse clustering persisted in dark-reared animals. We concluded that this data indicated that visual experience is critical for establishment of direction selectivity maps in the retina. The following year, it was demonstrated that DSGCs do not cluster along these cardinal axes as defined by motion in visual space, but rather the preferred directions of DSGCs cluster along axes defined apparent motion due to optic flow (Sabbah et al ,2017) Therefore, the distribution of preferred directions depends on the location on the retina. This direct

comparison of the data from the two studies indicates that the source of the errors in the previous study are due to combining preferred directions acquired from FOVs that ranged across nasal and temporal retina and undersampling. Indeed, the use of novel calcium dyes (Cal-590 and Cal-520) in this current study has greatly improved the sampling of DSGCs with significantly more DSGCs per FOV than in the previous study. This greatly improved dataset indicates that the direction selectivity map is well established at eye opening and not impacted by visual experience.

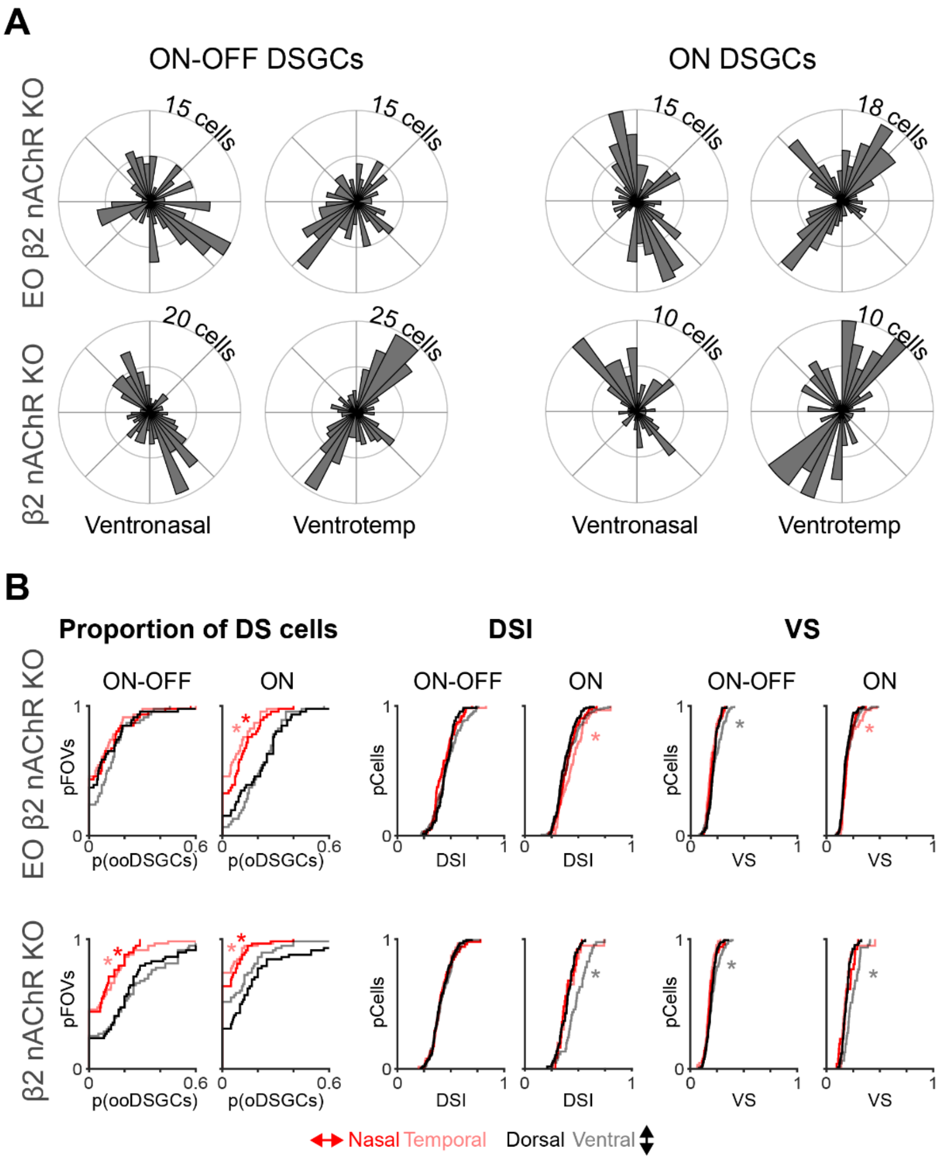

Figure S6. β2-nAChR-KO lack horizontal DSGCs in both ON and ON-OFF DSGCs, related to Figure 2.
A. Polar histograms depicting the DS maps of β2-nAChR-KO mice at eye opening (top) and adulthood (bottom) for ON-OFF and ON DSGCs in ventronasal and ventrotemporal retina.
B. Left: The proportion of field of views that exhibit different proportions of functional subtypes for ON-OFF and ON DSGCs of β2-nAChR-KO mice at eye opening (top) and adulthood (bottom). Middle & Right: same as left but for direction selectivity index (DSI) and vector sum (VS). * different from other experimental group, $p < 0.01$.
